# Modeling the structure-conditioned sequence landscape for large-scale protein design with TriFlow

**DOI:** 10.64898/2025.11.30.691458

**Published:** 2025-12-02

**Authors:** Harish Srinivasan, Rongqing Yuan, Qian Cong, Jian Zhou

**Affiliations:** 1 Department of Genetics Medicine, University of Chicago, Chicago, IL, USA.; 2 Lyda Hill Department of Bioinformatics, University of Texas Southwestern Medical Center; Dallas, Texas,USA.; 3 Eugene McDermott Center for Human Growth and Development, University of Texas Southwestern Medical Center, Dallas, TX, USA.; 4 Department of Biophysics, University of Texas Southwestern Medical Center, Dallas, TX, USA.; 5 Harold C. Simmons Comprehensive Cancer Center, University of Texas Southwestern Medical Center, Dallas, TX, USA.

## Abstract

Generative models have revolutionized computational protein design, and the design of high-quality sequences given backbone structure is a critical component for success. Current state-of-the-art design pipelines utilize sequence design methods with local structural context and autoregressive generation. To improve efficiency and quality of sequence design, we developed TriFlow, a model that combines a RoseTTAFold-like three-track architecture for global structural context with discrete flow-matching for efficient few-step sequence generation. We trained TriFlow on a large dataset of interacting protein chains from PDB and interacting domains from AlphaFold protein structure Database to enrich its knowledge of natural protein/domain interfaces. While showing improvements across diverse benchmarks, TriFlow’s primary advance is in de novo binder design, where it boosts the in silico success rate of state-of-the-art design pipelines such as BindCraft. We demonstrated this by conducting a large-scale benchmark, generating and validating binders for over 500 diverse protein targets. By leveraging the model to explore the designed sequence landscape, we discovered that we can effectively highlight functional active sites, by contrasting constraints learned by the structure-conditioned model with natural evolutionary profiles. As a practical demonstration of its capabilities, we apply our pipeline to systematically design specific binders against human class I cytokines family, computationally optimizing for on-target affinity while minimizing off-target interactions, demonstrating that specificity also scales with inference time computational budget. TriFlow thus provides a robust framework for both large-scale protein engineering and for exploring the fundamental principles of the structure-conditioned sequence landscape.

## Introduction

Designing protein sequences that fold into desired three-dimensional structures is a central challenge in computational protein engineering. Modern deep learning based design pipelines typically consist of two stages: backbone generation and sequence design. In the first stage, backbone structures are designed using generative models such as RFdiffusion or hallucination-based approaches like ColabDesign^1,2^. In the second stage, structure-conditioned sequence design models assign amino acids compatible with the target backbone. Finally, structure-prediction tools such as AlphaFold are used to refold the designed sequences and retain only those whose predicted structures closely match the intended backbone^3^.

Recent advances have produced a growing family of sequence design methods. They can be categorized by their core architectures as message-passing neural networks (MPNNs) such as ProteinMPNN and PiFold, transformer-based architectures such as ESM-IF, and physics-based methods such as Rosetta^4–6^. Among these, MPNNs have historically achieved strong performance when deployed to protein design pipelines, yet their local receptive fields limit their ability to capture long-range structural dependencies.

To overcome these limitations, we developed TriFlow to effectively capture global dependencies across the entire protein or a protein complex by combining advances in generative AI based on discrete flow matching and a new global attention architecture. The three-track global attention architecture is inspired by AlphaFold2 and RoseTTAFold2^3,7^. With discrete flow matching, TriFlow models the global structural context and decodes multiple residues simultaneously in each step, enabling the design of the entire protein sequence in as few as ten inference steps, regardless of protein size^8^. Trained on Protein Data Bank (PDB) structures using the same data splits as ProteinMPNN, TriFlow achieves superior performance across both monomer and binder design benchmarks. Moreover, we scaled the model training by creating a larger dataset, including interacting domain pairs in the AlphaFold protein structure Database (AFDB), to improve design accuracy and generalization^9^.

Leveraging the efficiency and robust performance of TriFlow, we conducted several large-scale in silico experiments to gain insights into the quality, specificity, and properties of designed sequences. We performed a large-scale in silico binder design campaign across more than 500 randomly selected protein targets. TriFlow consistently outperformed ProteinMPNN as a drop-in replacement in state-of-the-art design pipelines. Our analysis also uncovered factors influencing binder design success, including the chemical properties and secondary structures of interface residues, as well as the size of the endogenous interface.

We further examined the specificity of designed binders and attempted to generate selective binders for every human class I cytokine. Although off-target interactions were prevalent, we found that they can be substantially reduced by in silico selections of designed sequences against undesired binding. The designed specific binder sequences for human class I cytokines are released as a public resource to support potential biomedical applications.

Finally, we designed sequences for a set of 44k diverse domains representing all known homologous folds in Evolutionary Classification of protein Domains (ECOD)^10^. Comparing the designed and natural sequence profiles showed the largest disparities at protein active sites and small-molecule binding pockets. These findings point to a new use of structure-conditioned sequence design for large-scale functional annotation and suggest that jointly designing structure and function will be an important future challenge in protein engineering.

## Results

### TriFlow: modeling global context with discrete flow and global attention

**Figure 1.**
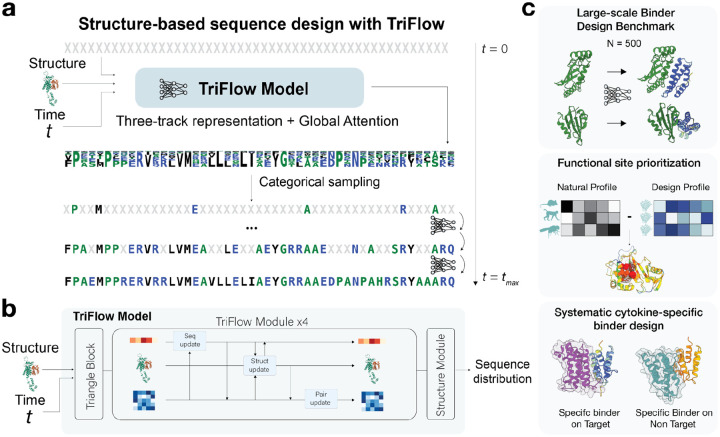
TriFlow overview. **a)** TriFlow sequence generation process starts with all masked tokens, and the model predicts a probability distribution for each AA position. These are sampled at each step to unmask the sequence until t_max. **b)** Key components in the TriFlow network (more details in supplementary Figure S1), which takes backbone structure, timestep, and sequence from a previous time step as input. **c)** Applications of our sequence design model in large-scale binder design benchmark, functional site prediction, and specific binder design for human cytokines.

To capture the full context of the protein structure for the sequence design problem, we combined discrete flow matching with a three-track evoformer-like architecture. Unlike autoregressive models such as MPNNs, discrete flow matching captures unconstrained global dependencies and enables parallel decoding of multiple amino acid positions, allowing for few-step sequence design for any backbone size (Fig. 1a). The three-track architecture utilizes transformers to integrate the single, pair, and 3D representations and capture both short and long-range interactions in proteins (Fig. 1b). Unlike the RoseTTAFold architecture we use invariant point attention and only use triangle multiplicative updates as opposed to the biased axial attention for the pairwise updates (supplementary Fig. S1).

As we anticipated that model performance would scale with training data size, we trained TriFlow on two datasets: a PDB dataset with the same train–test splits as ProteinMPNN to enable direct comparison, and an extended dataset specifically constructed to improve TriFlow’s binder-design performance and generalization. The extended dataset incorporates representative AlphaFold models from AFDB to better cover the breadth of protein sequence and structure space. In addition to PDB and AFDB monomer structures, we further included interacting PDB chains as well as interacting domain–domain pairs predicted by AFDB^11^ to strengthen TriFlow’s ability to design protein binders. To our knowledge, ESM-IF is the only structure-conditioned sequence design model trained on AFDB monomers, and no existing models have been trained on a predicted domain–domain interaction dataset. To compare performance between TriFlow and other methods, we used the TriFlow version trained on the ProteinMPNN training set, and for the large-scale binder design and other applications, we used the TriFlow version trained on this extended dataset.

It has previously been demonstrated that training the model with noise can be beneficial in protein design applications^4^. We extended this idea to train the model to handle multiple noise scales, allowing it to account for local uncertainty in the backbone coordinates. Introducing backbone noise appears to be essential when training with AFDB models, as we observed signs of overfitting when TriFlow was trained on unperturbed AFDB backbones. We extensively benchmarked TriFlow performance, including creating a new large-scale binder-design benchmark with 500 diverse target proteins, and demonstrated several novel applications of sequence design, including prediction of functional sites and designing specific binders for human class I cytokines (Fig. 1c, and details below).

### TriFlow improves protein sequence design

We performed comprehensive evaluations of TriFlow’s ability in sequence recovery and protein monomer design. First, we evaluated TriFlow’s ability to recover sequences for given structures using the shared PDB test set of 1392 structures between ProteinMPNN and TriFlow. TriFlow shows higher sequence recovery rate than previous methods, including ProteinMPNN, ESM-IF, and PiFold^4–6^. Second, to test TriFlow’s ability to design sequences for generated backbones, we used 3D structures with sequence lengths ranging from 100 to 1000, generated by hallucination method using the ColabDesign framework built on top of AlphaFold^12^. For each backbone size, we evaluated the refoldability of designed sequences, as indicated by the highest TM score between the target backbone structure and the predicted 3D structure of the designed sequence by AlphaFold3 in a single-sequence mode by disabling multiple sequence alignments (MSA) as input^13,14^. TriFlow, trained on the same training dataset as ProteinMPNN, outperforms all other models in sequence refoldability, particularly at the longer lengths, ranging from 600 to 1000 residues.

Interestingly, we found that introducing noise into backbone structures decreases TriFlow’s sequence recovery rate but increases the refoldability of the designed sequences (supplementary Fig. S2). This is consistent with previous models, such as ProteinMPNN. We hypothesize that introducing noise to backbone structures helps the model avoid overfitting to local errors in the generated backbones. Therefore, we observe better refoldability on the noisy backbones at longer lengths, since local errors can propagate.

We reasoned that TriFlow’s use of global structural context may enable it to more effectively capture dependencies between distant positions. Such inter-residue dependencies are typically reflected in amino acid covariance or coevolution signals, which in natural sequences arise from pairwise evolutionary constraints and closely mirror physical contacts within protein structures^15^. To evaluate whether structure conditioned sequence designed sequences satisfy similar coevolutionary constraints, we randomly selected 30 proteins from distinct clusters in the test set and generated 1,000 sequences for each. Using Gremlin, we quantified residue–residue “coevolution” and assessed whether residue pairs with the strongest coevolutionary signals (top L, L/2, and L/5 pairs) correspond to inter-residue contacts in the 3D structures.¹³ We compared TriFlow with ProteinMPNN and ESM-IF; PiFold was excluded because it deterministically produces only a single sequence per backbone and is therefore not suitable for this task.

**Figure 2.**
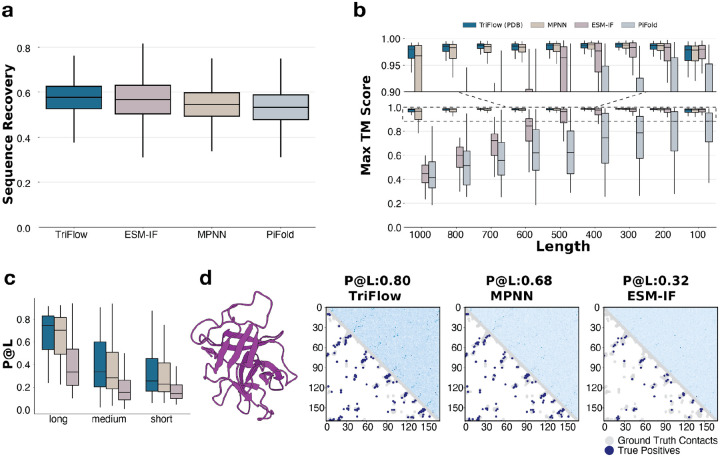
TriFlow outperforms previous tools in monomer sequence design. **a)** Native sequence recovery across different tools evaluated on the common test set used by ProteinMPNN and TriFlow, calculated as the fraction of residues for which the designed and natural sequences share the same amino acid. Potential train–test overlap cannot be excluded for ESM-IF and PiFold, which may slightly inflate their reported performance. **b)** Refoldability of sequences generated by different methods for a collection of ColabDesign-generated backbones of varying sizes (100–1000 residues).^12^ Refoldability is quantified as the maximal TM-score between the original backbone and the structures predicted by AlphaFold3 for the designed sequences. **c)** Agreement between the top-L coevolving residue pairs (L = protein length), inferred using Gremlin, and the inter-residue contacts in the native 3D structures. The analysis was performed on 30 representative proteins (clustered at 30% sequence identity) from the ProteinMPNN/TriFlow test set. Residue pairs were stratified by sequence separation: short (6 ≤ separation < 12), medium (12 ≤ separation < 24), and long (separation ≥ 24). **d)** Example protein structure (left) and the agreement between native contacts and the top-L coevolving residue pairs inferred from sequences designed by different tools. For each method, the upper triangle shows the coevolutionary couplings between residues inferred from the generated sequences, and the lower triangle shows the ground-truth contacts in the native structure. True positives which are residue pairs that appear both among the top-L coevolving pairs and in the ground-truth contacts are highlighted in blue, with all native contacts shown in grey.

We stratified inter-residue contacts by sequence separation into short, medium, and long-range categories. Across all categories, co-varying/evolving residues in TriFlow-designed sequences show the highest agreement with structural contacts (Fig. 2c). This suggests that TriFlow captures amino-acid dependencies more effectively than previous models, reflecting a stronger understanding of structure-derived pairwise constraints. Examining an illustrative example, ESM-IF-generated profiles display minimal consistency with contacts in the 3D structure (Fig. 2d). While both TriFlow and ProteinMPNN recover coevolution associated with 3D contacts, TriFlow demonstrates a closer alignment between its sequence profiles and the underlying structural constraints.

Another factor that could influence the coevolutionary signals in the generated sequences is the diversity of these sequences. This diversity is controlled by the *temperature* parameter, which determines the sharpness of the sampling distribution. The analyses above use the default temperature of 0.1 across all methods. We also ablated the temperature values from 0.1 to 1 to generate sequences of varying diversity and to observe their effects on amino acid dependencies (supplementary Fig. S3). All three models improved as temperature increased and achieved their best performance at a temperature at or near 1.0. Notably, because TriFlow’s sequence distribution recovers structural contacts more effectively even at lower temperatures, this property may also underlie its superior performance on other tasks, such as refoldability and sequence recovery.

### TriFlow improves state-of-the-art de novo binder design pipelines

**Figure 3.**
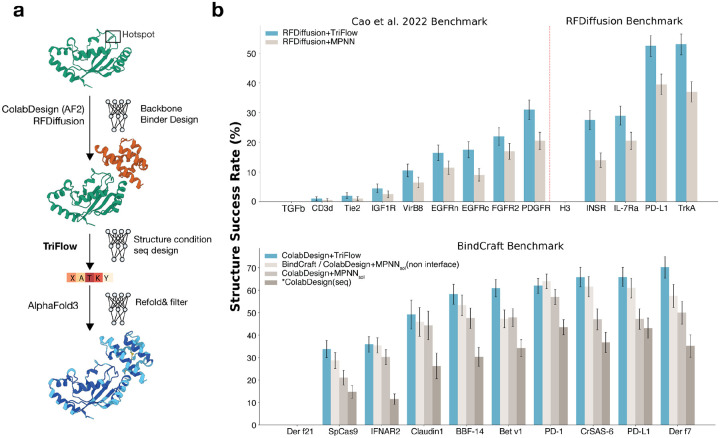
TriFlow outperforms ProteinMPNN and SolubleMPNN in protein binder design. **a)** Workflow describing the binder design pipeline: we first design backbones with RFdiffusion and ColabDesign, respectively, with binding hotspots specified for RFdiffusion; second, a set of sequences is generated with TriFlow for each binder backbone and then refolded with AlphaFold3. **b)** Performance of TriFlow and MPNN-based models evaluated by structure success rate, i.e., the percentage of binder backbones with at least one designed sequence that passed the established cutoffs based on AlphaFold3 confidence metrics. *Top:* Structure success rate on the combined set of therapeutic targets from a previous de novo binder-design study and the RFdiffusion paper. RFdiffusion+TriFlow: backbones generated by RFdiffusion with 8 sequences designed by TriFlow per backbone. RFdiffusion+MPNN: backbones generated by RFdiffusion with 8 sequences designed by ProteinMPNN per backbone. *Bottom:* Structure success rate on the BindCraft benchmark set. ColabDesign+TriFlow: backbones generated by ColabDesign with 8 sequences designed by TriFlow per backbone. BindCraft: backbones generated by ColabDesign with 8 sequences designed by SolubleMPNN per backbone, retaining the ColabDesign interface residues following the original BindCraft protocol. ColabDesign+MPNN_sol_: backbones generated by ColabDesign with 8 sequences designed by SolubleMPNN, including redesign of interface residues. *ColabDesign: both backbone and sequence generated by ColabDesign, which produces a single deterministic sequence per backbone and are often not used due to poor expression.

Targeting natural proteins with designed binders is a major application of de novo protein design. Binder-design pipelines typically follow four stages (Fig. 3a): selecting a hotspot or interface on the target (optional), generating a binder backbone conditioned on that site, designing a sequence compatible with the target–binder complex, and finally refolding the complex using a structure-prediction model such as AlphaFold3 in single-sequence mode with the target chain supplied as a template (Methods)^16,17^. Across nearly all contemporary pipelines, ProteinMPNN has been used as the standard model for sequence design.^1,2,12,18–20^ For backbone design, we benchmarked two most popular approaches, RFdiffusion, which designs binders using hotspots sampled from native interface, and ColabDesign, which does not require pre-defined hotspots to guide the structure generation process^1,2^.

To evaluate TriFlow in realistic de novo binder design settings, we benchmarked it against a panel of 24 therapeutically relevant protein targets from previous studies that used the RFdiffusion and BindCraft pipelines^1,2,21^. We first compared TriFlow’s sequence design ability against the original ProteinMPNN on RFdiffusion generated structures and a modified variant, SolubleMPNN (or MPNN_sol_), on BindCraft generated structures^4,18^.

We computed two key metrics to measure the strength of the sequence design methods. We assessed performance using **structure success rate**, defined as the percentage of designed binder structures that passed the established confidence thresholds (see Methods) after refolding with AlphaFold3. The **sequence success rate** is defined as the fraction of designed sequences that pass the same AlphaFold3 refolding criteria. We primarily relied on structure success rate because diversifying the scaffolds in the final pool of designed proteins are preferred in downstream experimental tests and sequence optimization^1^.

Across all 24 targets, we designed 8 sequences with each sequence design tool and evaluated them with AlphaFold3. TriFlow achieves a higher structure success rate (Fig. 3b) than either ProteinMPNN or SolubleMPNN. Three of the 24 targets (TGFβ, H3, and Der f21) could not be evaluated due to technical limitations in handling multi-chain templates in the AlphaFold3-based binder-evaluation script. Likewise, TriFlow outperforms both ProteinMPNN and SolubleMPNN in sequence design success rate (see Methods) for 19 of the 21 targets (supplementary Fig. S4 and S5). The only two exceptions are Claudin1 and PD-1 (supplementary Fig. S5), for which all three methods yield comparable performance. Together, these results demonstrate that TriFlow consistently generates higher-quality binder sequences than ProteinMPNN.

Next, we assess the performance of TriFlow in replacing SolubleMPNN within the widely used BindCraft pipeline, which currently represents the state of the art in binder design. BindCraft combines ColabDesign with SolubleMPNN. ColabDesign designs binder structures by optimizing sequences with back-propagation through the AlphaFold2 or AlphaFold-multimer networks, and thus these sequences are directly optimized for AlphaFold confidence metrics^1,22^. However, although ColabDesign-generated sequences show high AlphaFold3 confidence (supplementary Fig. S5), they often suffer from poor expression and solubility in experimental settings because the optimization is only based on structure loss and does not take the necessary sequence distribution such as solvent accessibility surface, charge distribution, etc. Subsequent redesign with SolubleMPNN has been shown to substantially improve experimental success rates^1,18^. Preserving interface residues designed by ColabDesign while redesigning only the non-interface residues with SolubleMPNN further boosts the structural success rate.

Replacing SolubleMPNN with TriFlow improved the structure success rate for all but one target (PD-1) in the BindCraft benchmark (Fig. 3b, **bottom**). Notably, this improvement does not depend on retaining the interface residues originally designed by ColabDesign and optimized for AlphaFold2 confidence metrics. In fact, redesigning **all** residues including interface positions with TriFlow yielded the highest structure success rate (supplementary Fig. S5), as measured by AlphaFold3 refoldability. This result indicates that TriFlow’s interface design is as effective as direct optimization through AlphaFold2.

### Large-scale binder design benchmark

To comprehensively evaluate model performance across a broad spectrum of protein targets, we constructed a large-scale binder-design (LBD) benchmark consisting of 500 diverse binary complexes randomly sampled from PDB entries with low (< 30%) sequence identity to proteins in the shared TriFlow–ProteinMPNN training dataset. This benchmark was designed to evaluate how TriFlow performs, instead of MPNN-based networks, within two widely adopted binder-design pipelines: (1) RFdiffusion → ProteinMPNN and (2) ColabDesign → SolubleMPNN (i.e., the BindCraft pipeline).

For each target, we generated 25 binder backbones using RFdiffusion and up to 10 backbones using a modified version of BindCraft to reduce compute cost which we call ColabDesign (see Methods). For each RFdiffusion-generated backbone, we designed 8 sequences using TriFlow and 8 using ProteinMPNN. For each ColabDesign-generated backbone, we designed 8 sequences using TriFlow and 8 using SolubleMPNN; TriFlow redesigned all residues, whereas SolubleMPNN redesigned only non-interface positions. In total, this benchmark comprises 16,216 binder backbones (12,500 from RFdiffusion and 3,716 from ColabDesign) and 129,728 designed sequences.

**Figure 4.**
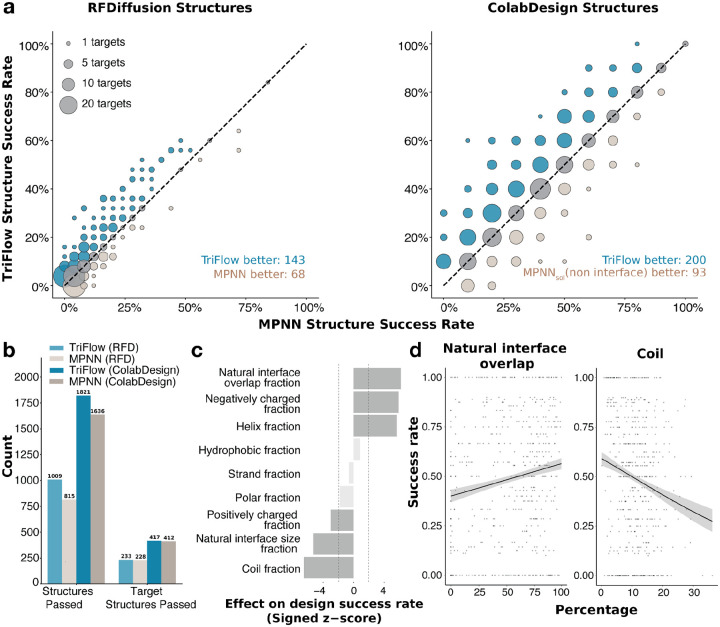
Large-scale benchmark of binder design pipelines. **a)** Comparison of structure success rates by different pipelines on 500 target structures. Left: binder backbones generated by RFdiffusion and 8 sequences designed by TriFlow and ProteinMPNN, respectively, for each backbone. Right: binder backbones generated by ColabDesign and 8 sequences designed by TriFlow and SolubleMPNN, respectively, for each backbone. **b)** Summary of the total number of binders that pass the in silico evaluation criteria (see Methods) for each pipeline, along with the number of targets for which at least one successful binder is obtained. In the labels above, the structure design method is indicated in parentheses and the sequence design method is outside the parentheses; RFD denotes RFdiffusion. **c)** Z-scores of logistic-regression coefficients quantifying the association between target features and design success. Positive values indicate that higher feature values are associated with increased success rates, and negative values indicate that higher feature feature values decrease success rate. **d)** Scatterplots with logistic regression fits showing the relationship between design structure success rate and two target properties: (i) interface overlap between the designed and natural binders, and (ii) the proportion of coil residues at the target interface engaged by the designed binders.

Across the entire LBD benchmark, TriFlow consistently outperforms ProteinMPNN and SolubleMPNN. For RFdiffusion-generated backbones, TriFlow achieves a higher structure success rate in 143 targets, whereas ProteinMPNN performs better on only 68 (Fig. 4a **left**). For ColabDesign-generated backbones, TriFlow performs better on 200 targets, while SolubleMPNN outperforms TriFlow on only 93 (Fig. 4a **right**). It is important to note that we designed only 8 sequences per backbone for the LBD benchmark, meaning the measured success rates for individual targets are subject to stochastic variability. We expect TriFlow’s advantage to become even more pronounced when more sequences per backbone are generated, as observed in our earlier small-scale benchmarks (Fig. 3b).

Both sequence designers perform better on ColabDesign backbones, yielding successful designs for 1,636/1,821 ColabDesign backbones across 412/417 targets, compared with 815/1,009 RFdiffusion backbones across 228/233 targets. (Fig. 4b). We compared structural properties of binder scaffolds with successful designs generated by RFdiffusion versus ColabDesign. Since RFdiffusion is guided by the native hotspots, it generates binders much closer to the native interface with a larger number of interface residues compared to ColabDesign (supplementary Fig. S7b,c). In our setup, ColabDesign is not specified with hotspots during generation, yet it tends to bind to the same native interface residues (supplementary Fig. S7a). Moreover, compared with native binding partners, binders generated by RFdiffusion and ColabDesign tend to contain more interface residues. Both structure-generation tools predominantly produce α-helical scaffolds with minimal β-sheet content; however, ColabDesign-generated binders exhibit a higher proportion of coil regions than those produced by RFdiffusion (supplementary Fig. S8).

To better understand which target properties influence sequence-design success, we leveraged our LBD benchmark to correlate diverse target features with design performance using the ColabDesign → TriFlow pipeline. We found that information about the native (natural) binding interfaces on a target are useful in guiding protein design strategies. These interfaces, shaped by evolutionary pressure, tend to have the physicochemical and geometric properties that are inherently suitable for binding^23^. Therefore, we observed that greater overlap between the designed binder interface and the native interface on the target correlates with higher success rates (Fig. 4c and 4d **left**). This observation supports the widely used strategy of anchoring de novo binders at native binding sites. Additionally, we found that the native interface size negatively correlates with design success (Fig. 4c). This effect is possibly an artifact due to the binder-size constraint imposed during backbone generation (60–120 residues). For targets with large native interfaces, the designed binders are likely too small to occupy the full binding site, making it more difficult for AlphaFold3 to identify a stable, correct binding mode for the designed complex. To improve binder design success rate, it might be important to scale binder size with the native interface size.

In addition, we found that properties of the target interfaces engaged by the designed binders are also predictive of design success. First, target interfaces with higher α-helical content are associated with higher success rates, whereas interfaces enriched in coil regions show lower success (Fig. 4c, Fig. 4d **right,** and Supplementary Fig S9). This likely reflects the fact that designed binders are themselves highly α-helical, making them more compatible with helical interface geometries. Second, target interfaces with a larger proportion of negatively charged residues are associated with greater success, whereas interfaces enriched in positively charged residues tend to be more challenging (Fig. 4c). We reason that this trend may also arise from the helical nature of the designed binders: positively charged residues (Arg and Lys) have strong helical propensities, whereas one of the negatively charged residue Asp is a well-known helix breaker^24,25^. Thus, while designing helical binders with positively charged surfaces to bind negatively charged interfaces is relatively straightforward, designing helical binders that present negatively charged surfaces to engage positively charged target interfaces may be more difficult. Finally, we observed a modest positive correlation between interface hydrophobicity and design success. Because native protein–protein interfaces typically feature hydrophobic residues at the core and hydrophilic residues at the periphery, the relationship between hydrophobicity and successful design is likely more nuanced than a simple monotonic trend^26^.

### Designing specific binders for all class I cytokines

While prior de novo design studies have successfully generated binders for individual targets, few have developed systematic pipelines to evaluate and minimize off-target binding to closely related proteins. To explore possible strategies to design highly specific binders, we applied the best-performing binder design pipeline identified from previous benchmarks to the human class I cytokines, which comprises 31 homologous proteins. Class I cytokines are four-α-helix bundle signaling proteins that act through WSXWS-containing hematopoietin receptors to regulate immunity, development, and hematopoiesis^27^.

Class I cytokines are major therapeutic targets, and their dysregulation contributes directly to autoimmune diseases, inflammation, immunodeficiency, cancer, and blood disorders^28^. Existing therapeutics for Class I cytokines are predominantly protein-based agents that bind the cytokines and prevent them from engaging their receptors and initiating downstream signaling^29^. Because Class I cytokines act extracellularly, they are readily accessible to biologics without the challenges associated with intracellular delivery^29^. These properties make Class I cytokines particularly attractive targets for de novo designed protein therapeutics.

Using Class I cytokines as an example, we set out to evaluate the target-specificity of de novo designed binders by modern pipelines. For each cytokine target, we generated up to 10 backbone structures using ColabDesign, designed 40 sequences per binder with TriFlow, and screened with AlphaFold3 to identify highly confident de novo binders. We were able to obtain binders for all cytokine targets except for IL7.

**Figure 5.**
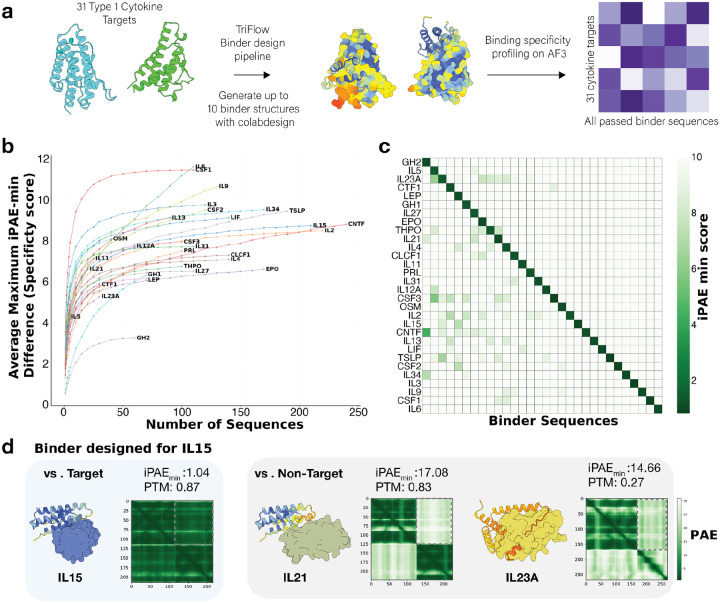
Systematic design of specific binders for class I cytokines. **a)** Overview of the pipeline used to generate cytokine-specific binders. On top of the standard “ColabDesign → TriFlow → AlphaFold3” pipeline, we added all-against-all binding evaluation between designed binders and cytokines to evaluate the off-target effects. **b)** Post hoc analysis showing the scaling relationship between predicted binder specificity and the number of binder sequences designed for each target. Specificity is quantified as the difference in ipAE _i_ between the intended target and the best off-target, and this value is the average for 100 random samples of a certain number of designed sequences to minimize the random effects. **c)** Heatmap depicting on-target and off-target interactions for each selected binder (with optimal specificity score) based on ipAE _i_ scores, highlighting the specificity achieved for each cytokine binder. The designed binders are ordered from the left to the right on the X-axis by decreasing specificity score, and their corresponding targets are ordered from the top to the bottom on the Y-axis. **d)** Example of a designed binder targeting IL15, which shows a low on-target ipAE _i_ (1.04), poor off-target interaction with IL21 (high ipAE _i_), and unfavorable structure prediction for IL23A (low predicted TM-score for the binder).

To evaluate the off-target effect of each designed binder, we used AlphaFold3 to model its interaction with every class I cytokine (Fig. 5a). To monitor such off-target effects, we quantified the specificity of a designed binder using a specificity score, defined as the difference between the lowest predicted ipAE _i_ (on-target) and the best off-target ipAE _i_ value (see Methods). For most cytokines, we successfully designed multiple specific binders with high specificity scores (supplementary Fig. S10c).

We reasoned that we can optimize target specificity and minimize off-target effects by designing more sequences for each backbone with TriFlow and selecting binders with the highest specificity scores, leveraging TriFlow’s fast inference speed designing in seconds for each sequence for TriFlow versus hours per backbone for ColabDesign). As expected, the best specificity score for each target increased as more binders were designed (Fig. 5b), with the most substantial gains observed when increasing the number of sequences from 1 to 30 per target. Beyond 30 sequences, specificity scores begin to plateau for most cytokines; however, for certain targets such as IL-6 and EPO, specificity can continue to improve with additional TriFlow-designed sequences. With this simple scaling strategy, binders for most cytokines reach high specificity scores. In contrast, some targets, particularly GH2, plateau at more moderate specificity, suggesting that achieving high specificity for these proteins may require additional structure generation or selecting alternative interface hotspots.

We designed 4–241 sequences for each target (Supplementary Fig S10c). After picking the binder sequence with the best specificity score for each target, we analyzed the ipAE _i_ for each selected binder against every target (Fig. 5c). Those with lower specificity scores tend to show more and stronger off-target interactions. While our optimization of specificity scores increases the gap between on-target ipAE _i_ and off-target ipAE _i_ scores, the gap between on-target and off-target ipTM scores is also prominent (supplementary Fig. S10b). We examined the predicted on- and off-target interactions for the binder with the highest specificity score for IL15 (Fig. 5d). The binder is predicted to bind IL15 with high confidence, while its interactions with off-targets such as IL21 and IL23A show low confidence. In the IL23A example, the binder not only fails to form a confident complex but also does not fold into its intended structure, providing an additional indication of poor off-target binding.

### Designed sequence profiles informing functional site identification

Evolutionary constraints, which can be inferred from multiple sequence alignments (MSA) across natural protein homologs, have long been essential for inferring protein structure and function^30^. Because TriFlow can generate diverse sequences conditioned solely on a target structure, we sought to ask what are the similarities and differences between designed and natural sequence profiles, and to assess whether their differences could help disentangle the relative contributions of structural versus functional constraints in protein evolution. To this end, we designed sequences for 43,659 diverse protein domains spanning all major evolutionary groups represented in the Evolutionary Classification of Domains (ECOD) database^10^.

**Figure 6.**
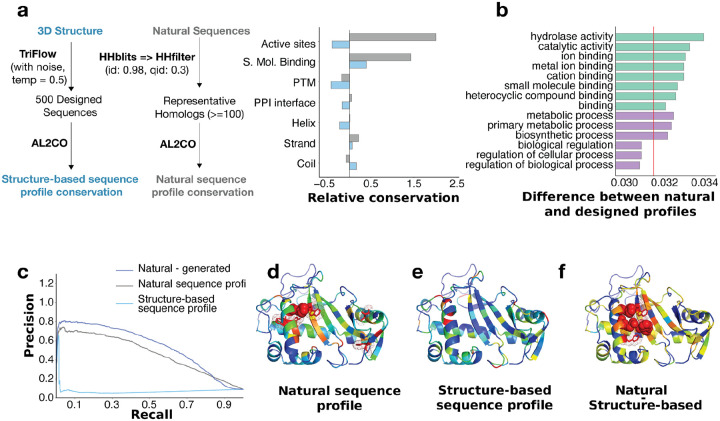
Functional site prediction with TriFlow-designed profiles. **a)** A pipeline to generate designed (left) and natural (mid) sequence profiles and compute sequence conservation. The right plot shows the relative conservation (compared to the average over all residues) of different residue types in natural and designed sequence profiles. **b)** Common Gene Ontology (GO) terms (>1,000 proteins) showing significant differences between natural and designed profiles. Such differences are quantified by Wasserstein Distance at each position, averaged across proteins. The red line indicates the average distance between natural and designed profile over all positions in all proteins. Green indicates molecular function terms and purple indicates biological process terms. **c)** Precision and recall curves at a positive-to-negative ratio of 1:10 for functional site (active site and small molecule binding site) prediction based on natural profile, designed profile, or the difference between natural and designed profiles. **d-f)** 3D structure of a protein colored by conservation in **d)** natural profile, **e)** designed profile, **f)** the difference between natural and designed profile.

We compared conservation patterns in natural sequence profiles with those in the TriFlow-designed profiles for the same domains (Fig. 6a). The largest discrepancies occur at functional sites, particularly active sites (mainly catalytic enzyme residues) and small-molecule binding sites annotated in UniProt^31^. These positions are under strong purifying selection in evolution and therefore highly conserved in natural sequences, whereas such functional constraints are absent in structure-conditioned sequence design^32^. Accordingly, designed sequences show only moderate conservation at small-molecule binding residues and almost no conservation at active sites. This likely reflects the fact that small-molecule binding pockets often require unusual or precise structural features, whereas catalytic residues impart relatively little constraint on the 3D structures. Interestingly, post-translational modification (PTM) sites are poorly conserved in both natural and designed profiles, consistent with the observation that PTM positions are not strongly conserved across species^33^. Moreover, most PTMs occur in flexible, solvent-exposed regions that impose minimal structural constraints, explaining their low conservation^33^.

In contrast to functional sites, loops and coils are more conserved in designed sequences than in natural sequences. In natural proteins, these regions are structurally flexible and tolerate extensive sequence variation.^34^ However, because current design pipelines assume a rigid backbone, even in loops and coils, these positions experience artificially strong structural constraints that lead to elevated conservation in designed profiles.

Consistent with these residue-level observations, we also found that at the whole-protein level enzymes and small-molecule–binding proteins show the largest divergence between natural and designed profiles (Fig. 6b). In contrast, proteins that function primarily through protein-protein interactions (PPIs), such as regulatory proteins, display relatively small differences between natural and designed sequences.

The pronounced differences between natural and designed sequence profiles at functional sites suggest a new strategy for identifying residues under strong functional constraints. By subtracting the conservation in designed sequences from that in natural sequences, we can effectively remove the contribution of structural constraints and highlight positions that are selectively intolerant to mutation due to functional constraints. This simple approach markedly improves the precision–recall relationship for distinguishing true functional sites from background positions (blue versus grey curves in Fig. 6c). An illustrative example, the fungal ribonuclease Rh, is shown in Fig. 6d and Fig. 6e, where the 3D structure is colored from red to blue according to decreasing conservation. In the natural profile, only one of the five most conserved residues corresponds to a catalytic site (red sphere), and another lies adjacent to this active residue facing the substrate-binding pocket; the remaining highly conserved positions likely reflect structural constraints. In contrast, the most conserved residues in the designed profile track unusual structural features and frequently occur in flexible loops. Notably, the difference between natural and designed conservation indices more effectively highlights functional residues: the positions with the largest conservation differences correspond either directly to active-site residues (shown as red spheres in Fig. 6f) or to neighboring positions lining the substrate-binding groove (shown as red sticks in Fig. 6f).

## Discussion

We presented a comprehensive exploration of structure-conditioned sequence design space that spans generative methodology, model architecture, training data, benchmarks, and applications. TriFlow advances structure-conditioned protein sequence design by unifying a global three-track RoseTTAFold/AlphaFold-like architecture with discrete flow matching. In contrast to autoregressive MPNN-based approaches, TriFlow leverages global structural context and parallel decoding to capture long-range dependencies across the entire protein or complex, enabling efficient few-step sequence generation for backbones of any length. Our results show that this architectural shift leads to consistent gains in both monomer and binder design performance.

In the monomer design setting, TriFlow outperforms existing sequence-design methods in both native sequence recovery and refoldability. The advantage is particularly pronounced for large backbones, where long-range structural constraints are critical. Introducing backbone noise during training reduces exact sequence recovery but improves refoldability, suggesting that TriFlow learns to produce sequences that are robust to local structural perturbations rather than memorizing native residue identities. The use of Evoformer-like pair representations helps TriFlow to learn dependencies between residues and use it to guide sequence generation. TriFlow-designed MSAs recover residue-residue couplings that align more closely with 3D contacts than those generated by other tools. Together, these findings indicate that TriFlow learns a sequence distribution that encodes global structural constraints more faithfully than prior models.

A major motivation for developing TriFlow was to improve de novo binder design. Across both a curated set of therapeutic targets and a new large-scale benchmark, TriFlow consistently outperforms ProteinMPNN and SolubleMPNN as a drop-in replacement in state-of-the-art pipelines based on RFdiffusion and BindCraft. In the BindCraft pipeline, SolubleMPNN was only used to redesign the non-interface residues because keeping the interface residues designed by ColabDesign increases the pipeline’s success rate. In contrast, TriFlow improves structure success rates not only when redesigning non-interface residues but also when redesigning the entire binder. This suggests that TriFlow can match the interface optimization achieved by backpropagation through AlphaFold2, despite being trained as a standalone sequence generation model. One plausible explanation is that TriFlow’s AlphaFold-inspired architecture enables it to internalize global structural priors similar to those used by AlphaFold, producing sequences that are intrinsically favored by AlphaFold3. Yet, as a generative model, TriFlow can design diverse sequences much more efficiently than backpropagation through AlphaFold. It is important to note that our in silico evaluations rely on AlphaFold as surrogates for experimental success. While this is a common practice in the field, experimental validation will be essential to quantify how often improvements in AlphaFold-based metrics translate into real-world gains.

In addition to the discrete flow and global attention-based model design, we expanded the training dataset to include interacting domain pairs from AFDB alongside PDB structures, which exposes the model to more diverse protein interfaces. While the expanded training dataset is expected to benefit the research community, we also observed that potential inaccuracies in AlphaFold-predicted structures can adversely affect model performance. For example, when backbone noise is not assumed during training, incorporating additional AFDB models leads to a slight decrease in performance, likely reflecting the impact of errors or systematic biases in predicted structures.

For robust benchmarking of computational binder design pipelines, we introduced a large and diverse 500-target binder design benchmark (LBD). LBD is more than an order of magnitude larger than prior benchmarks, and we hope it will facilitate better evaluation of binder design methods. With these large-scale design results, we systematically analyzed which target and interface properties predict binder design success rate, which provide guidance to applications and directions for improvement for future method development. We found that helical, electrostatically favorable target interfaces depleted of positively charged residues tend to represent easier design cases for modern protein-design pipelines. For targets whose interface properties are associated with lower success rates, generating larger numbers of designs can help compensate for reduced performance. We also confirmed that engaging the native binding interface on the target increases design success, and that the size of the binder should scale with the size of the native interface to fully occupy the binding surface that has been evolutionarily optimized for PPIs.

Specificity is an important yet understudied dimension of de novo binder design. In our design campaign targeting all class I cytokines, we found that although most binders exhibit strong predicted preference for their intended targets, off-target interactions remain non-negligible. Nevertheless, we showed that specificity can be improved by scaling up the number of designed sequences and explicitly evaluating cross-reactivity across related targets. We provide computationally predicted specific binders for most class I cytokines as a public resource. These designs offer a valuable starting point for experimental validation and illustrate how efficient sequence generators like TriFlow can accelerate the development of highly specific protein binders.

By systematically comparing generated structure-conditioned sequence profiles versus natural sequence profiles, we discovered a lack of constraints on functional sites in generated profiles. Compared to natural sequence profiles, MSAs of designed proteins show markedly reduced conservation at enzyme active sites and small-molecule binding pockets, consistent with the idea that many catalytic residues and ligand-coordinating positions exert relatively weak constraints on the global fold. Subtracting conservation in designed sequences from natural conservation values enhances functional-site prediction relative to using natural profiles alone, highlighting the potential of structure-conditioned sequence design models to aid in interpreting and understanding natural protein function.

Finally, our analyses also highlight several limitations and corresponding opportunities for advancing TriFlow and structure-conditioned sequence design. First, current models optimize sequences solely for structural compatibility, without incorporating functional requirements; integrating explicit functional objectives, such as catalytic efficiency or ligand specificity, into training or sampling could enable true joint structure-function design. Second, existing pipelines assume rigid backbones even in loops and coils, producing artificially high conservation in flexible regions; incorporating backbone ensembles, conformational dynamics, or explicit flexibility during design would help better capture natural variation. Third, sequence design and structure generation are currently performed separately, limiting the ability to co-optimize both components; coupling TriFlow with generative structure models offers a way to jointly refine backbone and sequence for improved structural and functional performance. Finally, the designed binders produced by established pipelines are overwhelmingly α-helical, whereas natural PPI interfaces frequently rely on diverse structural motifs, including β-strands, mixed secondary structures, and irregular loops, to achieve high affinity and specificity. This bias arises not only from the structure-generation tools but also from the fact that α-helical scaffolds are more readily folded and confidently predicted by AlphaFold, even without MSAs. Overcoming this limitation will likely require a new generation of structure-prediction and backbone-generation models that can more faithfully capture the diversity of natural folds and PPI interfaces.

In summary, we expect TriFlow to be widely applicable across various protein design tasks and beyond. To that end, we provided an open-source repository with user-friendly tutorials to facilitate usage across the community.

## Materials and Methods

### Model Architecture

The TriFlow architecture follows the design of modern protein structure predictors, where multiple iterative attention-based blocks update single, pair, and 3D rigid frames of residues in a protein that exchange information across representations. Single representations are updated using gated attention with pair bias, allowing pair features to modulate attention weights. 3D coordinate updates combine invariant point attention with backbone transformations to maintain rotational and translational consistency. Pair representations are refined through triangle multiplicative updates, like in AlphaFold’s Evoformer, to capture higher-order residue interactions.

Before the main TriFlow blocks, we run a single triangle block comprising triangle multiplicative updates and triangle attention. We use triangle attention in addition to triangle multiplicative update, as it has been shown to be important, especially for structure prediction, in constructing dense 2D distance embeddings. Because triangle attention is expensive and the backbone does not change across timesteps, we compute the triangle block once at the start, cache the resulting pair embeddings, and reuse them for all sampling steps. This reduces computational cost by running the triangle block once rather than running it independently at all 10 sampling steps.

### Training

We trained a version of TriFlow on the same dataset as ProteinMPNN, which are protein assemblies in the PDB until August 2021, clustered at 30% sequence identity and split into train, validation and test sets, ensuring assemblies belonging to different sets never share proteins belonging to the same cluster. We additionally trained a version of TriFlow with additional representative entries from AFDB and predicted domain-domain interactions from AFDB models to enrich interface design.

The model was initially trained with a crop size of 256 residues and later with 512 residues, using spatial crops centered at the interface in 50% of the samples and contiguous segments in the remaining samples. During training, the backbone input was left unperturbed in half of the samples, while in the other half it was corrupted with Gaussian noise (standard deviation = 0.2 Å) to improve robustness. In addition, the model is provided with a noise token, which lets it know if the backbones are perturbed. We trained the final TriFlow model on AFDB using two backbone noise settings. Half of all training steps used a low noise level of 0.02 Å, and the remaining half used a higher noise level of 0.2 Å. At each step, the backbone was perturbed with one of these levels sampled with equal probability.

Training followed a discrete flow-matching objective that linearly interpolates between the masked state, an extra state beyond the 20 canonical amino acids, and the native sequence along a continuous-time Markov chain trajectory. We specifically follow discrete flow models implementation for training and sampling (supplementary Fig. S11 and Fig. S12). The model was optimized using cross-entropy loss between the predicted logits and the true one-hot-encoded amino acid sequence.

We use the AdamW optimizer with a learning rate warmup of 1000 steps from 0 to 0.001. We train the model with a batch size of 8 per GPU on 4 A100 GPUs. For the TriFlow model trained on both PDB and AFDB data, we initialized the model weights with the pre-trained weights from the model trained on ProteinMPNN training dataset.

### Sequence Recovery, Refoldability and Residue-Residue Interaction Analysis

When evaluating sequence recovery, we filtered the ProteinMPNN test set to include only PDB biological assemblies with fewer than 1000 residues and randomly sampled one assembly per cluster. We compared TriFlow trained on ProteinMPNN train set, ProteinMPNN trained with 0.02 noise, ESM-IF and PiFold. To assemble the refoldability benchmark, we used the pregenerated structures with relaxed sequence optimization (RSO) in ColabDesign from a previous study.^12^ We sampled eight sequences per structure and compared TriFlow with 0.2Å noised backbones trained on the ProteinMPNN dataset to ProteinMPNN with 0.2 Å noise, ESM-IF, and PiFold. For all models, we used a sampling temperature of 0.1, except for PiFold, for which we always picked the amino acid with maximal probability following the authors’ recommendation. We used AlphaFold3 in single-sequence mode, without MSAs or templates as inputs.

We assessed whether the backbone-conditioned sequence design distribution reflected explicit structural constraints and whether the model learned dependencies (coevolution) between different positions. We randomly selected 30 biological assemblies (one or multiple PDB chains) in the ProteinMPNN test set. We generated 1000 sequences for each biological assembly by three methods, including TriFlow, ProteinMPNN trained on 0.2 Å noise, and ESM-IF, to create the generated profiles. To check whether the generated profiles contained information about structural constraints, we used Gremlin to identify coevolving residues. We ablated across temperature sampling from 0.1 to 1 for all tools, and also used backbones with or without noise for TriFlow.

### Binder Design Benchmarks

For the RFdiffusion benchmark, we curated target structures from prior papers (RFdiffusion paper and a previous Rosetta-based binder design work) and generated 200 structures with RFdiffusion. We designed eight sequences per backbone with TriFlow and ProteinMPNN, respectively. We specified the hotspots based on previous literature, otherwise we used hotspots randomly sampled from the binding interface used by previously successful de novo binders to the targets.

We ran the BindCraft pipeline on their own benchmark set, following the interface hotspot they specified (see Table S1). We ran the pipeline for 2 days for each target on one A100 GPU and used all the backbone structures it generated for our benchmarking.

A binder sequence was considered successful in silico if it met established criteria derived from AlphaFold2 and AlphaFold3 evaluations of the target-binder complex^35^. We followed the AlphaFold implementation with initial guess derived from the target structure and without MSAs (single-sequence mode) for the AlphaFold2 screening, and used the following cutoffs from the alphaproteo benchmarks to select successful binders: binder RMSD < 1.5, average pAE of interface residues < 7, and binder pLDDT > 90. For AlphaFold3, we use the target chain as a template and run the model in single-sequence mode (https://github.com/Kuhlman-Lab/AlphaFold3)^35,36^. We ran three rounds of recycling in AlphaFold3 and used the following cutoffs to select successful binders: ipAE _i_ < 1.5, binder pTM > 0.8, and complex RMSD < 2.5.

Because the AlphaFold3 wrapper script we obtained only supports single-chain target structures, and naively concatenating multiple chains in the target structure did not work. As a result, we did not get any binder sequence to pass the AlphaFold3 filter for the following multi-chain targets, H3 in the RFdiffusion benchmark, and Der f21 in the BindCraft benchmark.

### Large-scale Binder Design Benchmark

We collected 53,471 PDB entries released between August 2021 and April 2025, extracted 71,678 biological assemblies, and identified 955,925 interacting chain pairs in which each chain contained at least five residues within 5 Å of the other chain. To avoid overlap with the training data for ProteinMPNN and TriFlow, we excluded interacting pairs whose close homologs (detected by MMseqs at >30% sequence identity and >60% query or hit coverage) appeared in PDB entries released prior to August 2021. This filtering yielded 297,486 interacting PDB chains. We clustered these chains using MMseqs^37^ (50% identity, 80% coverage) and selected one representative pair per cluster-cluster combination, resulting in 15,367 representative interacting pairs. Most of these pairs originate from higher-order complexes, for which disrupting native assemblies using designed binders might be more challenging. We therefore restricted our focus to the 772 representative pairs derived from binary complexes and randomly selected 500 of them to construct the LBD benchmark. For each binary complex, we randomly designated one chain as the target and used the native binding interface to guide hotspot selection for the RFdiffusion-based pipeline.

We generated binder backbones using both RFdiffusion and ColabDesign from the BindCraft pipeline. To generate binders with RFdiffusion, we provided the native hotspot and generated lengths ranging from 60 to 120 residues. As the BindCraft hallucination protocol is expensive, requiring 140 AlphaFold forward passes for a successful trajectory, we made some necessary modifications to save time and reduce compute cost. The BindCraft pipeline consists of structure generation by ColabDesign, sequence redesign by SolubleMPNN, and additional functionality, including Rosetta scripts and self-consistency with AlphaFold2. We took the ColabDesign-based structure generation modules from BindCraft and modified the script to generate structures up to a certain number of trials (specified by a user), rather than the number of successfully generated structures that pass filters. BindCraft would terminate unpromising trajectories if the binder pLDDT went below 65, and thus the number of generated structures could be below the number of trials. This makes the compute time spent on structure generation more predictable and easier to benchmark. We used this ColabDesign script without any hotspots to generate up to 10 backbone structures for each target, requiring each backbone to contain 60–120 residues.

For each backbone generated by RFdiffusion, we performed sequence design with ProteinMPNN and TriFlow, respectively, generating 8 sequences for each backbone. For backbones generated by BindCraft/ColabDesign, we replaced ProteinMPNN with SolubleMPNN from the BindCraft pipeline for comparison against TriFlow. We used TriFlow trained on PDB (prior to August 2021) and AFDB with 0.2 Å noise added to the backbone and set its temperature to 0.1. Similarly, we used ProteinMPNN and SolubleMPNN trained with 0.2Å noise and set the temperature to 0.1. The BindCraft pipeline by default only redesign the non-interface residues by SolubleMPNN, leaving the interface residues designed by ColaDesign. To test the impact of this strategy, we attempted to redesign all residues or only focus on non-interface residues. We also tried to assume backbone noise (0.2Å standard deviation) in the BindCraft-generated structures and tested whether this assumption affected TriFlow’s performance. During sequence design for all setups, we omitted cysteine during the sequence generation.

### Design Specific Binders for Class I Cytokines

We gathered the predicted 3D structures for 31 human Class I cytokines from AFDB and removed their signal peptides following UniProt’s annotations. We initialized 10 binder backbone generation trials for each cytokine with ColabDesign. However, a fraction of these trials would be automatically terminated due to the pLDDT-based checkpoint (see previous section about our modifications to the BindCraft script), resulting in 2–8 successfully generated backbone structures for each cytokine. We designed 40 sequences using TriFlow trained on both PDB and AFDB for each generated backbone structure. Across the 31 cytokine targets, all but one (IL7) yielded at least one binder passing the same AlphaFold3 thresholds we used in the binder design benchmarks (ipAE _i_ < 1.5, binder pTM > 0.8, and complex RMSD < 2.5).

To estimate the cross reactivity to other targets, we computed a specificity score, *S*_*spec*_, for each binder *x* intended target cytokine *T*_*t*_ based on the following formula:

$${\text{ipAE}}_{\text{min}}^{\text{on}}={\text{ipAE}}_{\text{min}}\left(x,T_{t}\right),$$

$${\text{ipAE}}_{\text{min}}^{\text{off}}=\underset{j\neq t}{\text{min}}\;{\text{ipAE}}_{\text{min}}\left(x,T_{j}\right),$$

$$S_{\text{spec}}={\text{ipAE}}_{\text{min}}^{\text{off}}-{\text{ipAE}}_{\text{min}}^{\text{on}},$$

where ipAE _i_ is the minimal interface pAE computed by AlphaFold3 and *T*_*j*_ is any Class I cytokine in our target set. We aimed to identify the binder with the highest *S*_*spec*_, while satisfying another criterion of binder pTM > 0.8 estimated by AlphaFold3, among our pool of designed binders for each cytokine target. These optimized sequences became our final set of designed binder sequences for human class I cytokines (Fig. 5c).

We conducted a post hoc analysis to examine the relationship between the maximal binder *S*_*spec*_ for each cytokine target and the number of sequences, *N*_*design*_, designed for each binder backbone for the cytokine target. To examine this relationship, we scanned different values of *N*_*design*_, randomly sampled *N*_*design*_ designed sequences for each binder backbone, and repeated the sampling 100 times to calculate the average maximal *S*_*spec*_ (Fig. 5b).

### Comparison of Natural and Designed Profiles and Functional Site Prediction

We clustered ECOD domains parsed from PDB structures using MMseqs (-c 0.8 --min-seq-id 0.4) and selected one representative domain per cluster. For each representative domain, we used its 3D structure as input to TriFlow trained with 0.2 Å coordinate noise, and generated 500 sequences at a sampling temperature of 0.5. Because all 500 sequences were designed for the exact same backbone, they were implicitly aligned and together constituted the designed sequence profile. We used a high temperature here to increase the diversity of designed sequences.

To construct natural MSAs, we searched for homologs of each domain sequence using HHblits^38,39^ against the UniRef30 database. The resulting MSAs were filtered with HHfilter^38,39^ to remove highly similar sequences (identity > 98%) and sequences that were too distantly related to the query (identity < 30%). These filtered MSAs served as the natural sequence profiles. Since some domains lacked sufficiently deep MSAs, we restricted downstream analyses to the 19,264 ECOD domains with at least 100 sequences in the filtered MSA.

We next annotated structural and functional properties for each residue in every ECOD domain. We downloaded UniProt’s reviewed entry annotations (JSON format) and mapped them to the corresponding PDB entries. This allowed us to annotate active sites (catalytic sites), small-molecule binding sites, and PTM sites (including modified residues, glycosylation, and lipidation). In total, 16,976 ECOD domains were mapped to reviewed UniProt entries. To identify PPI interface residues, we examined the original PDB structure for each domain and marked positions with any atom within 8 Å of another chain as PPI interface residues. Additionally, secondary structure assignments were obtained using DSSP^40^.

For each position in every domain, we computed conservation indices for both the natural and designed MSAs using AL2CO^41^. For each structural or functional category (as annotated above and shown in Fig. 6a), we averaged the conservation indices across all category-associated residues and compared these averages to the global averages across all residues in natural or designed profiles.

To quantify differences between natural and designed profiles at the domain level, we calculated a profile-divergence score for each ECOD domain by averaging the Wasserstein distance^42^ between the natural and designed conservation distributions over all positions. We denote the divergence for domain *i* as *PD*_*i*_. To classify domains into functional categories, we applied ProteInfer^43^ to predict Gene Ontology (GO) terms for each domain sequence. For each GO term, we compared the *PD*_*i*_ for domains annotated with that term against *PD*_*i*_ values for domains not annotated with that term. Statistical significance was assessed using the Mann-Whitney U test, followed by false discovery rate correction (Q-values)^44^ to account for multiple testing. We reported GO terms in Fig. 6b that met the following criteria: (1) associated with more than 1,000 domains; (2) Q-value < 0.05; (3) belonging to the “molecular function” or “biological process” categories.

The marked discrepancy in conservation at catalytic active sites between natural and designed profiles prompted us to test whether contrasting these profiles improves functional-site prediction. As positive controls, we used annotated active-site residues in the ECOD domains. Negative controls were all other residues not annotated as active sites, binding sites, or PTM sites in UniProt; the negative set was subsampled to 10× the size of the positive set. We then evaluated whether the two sets could be distinguished using three metrics computed per residue: (1) the AL2CO conservation index from the natural profile, (2) the conservation index from the designed profile, and (3) the difference between natural and designed conservation indices (Fig. 6c).

## Supplementary Material

1
