# Supplementary material for: Modeling the structure-conditioned sequence landscape for large-scale protein design with TriFlow
Source: bioRxiv. 2025 Dec 2:2025.11.30.691458. Preprint. [Version 1] doi: 10.64898/2025.11.30.691458 (PMC12707284; doi:10.64898/2025.11.30.691458)
Supplement: 1 [file NIHPP2025.11.30.691458V1-supplement-1.pdf]

bioRxiv preprint doi: https://doi.org/10.64898/2025.11.30.691458; this version posted December 2, 2025. The copyright holder for this preprint (which was not certified by peer review) is the author/funder, who has granted bioRxiv a license to display the preprint in perpetuity. It is made available under aCC-BY 4.0 International license.

Supplementary Figures

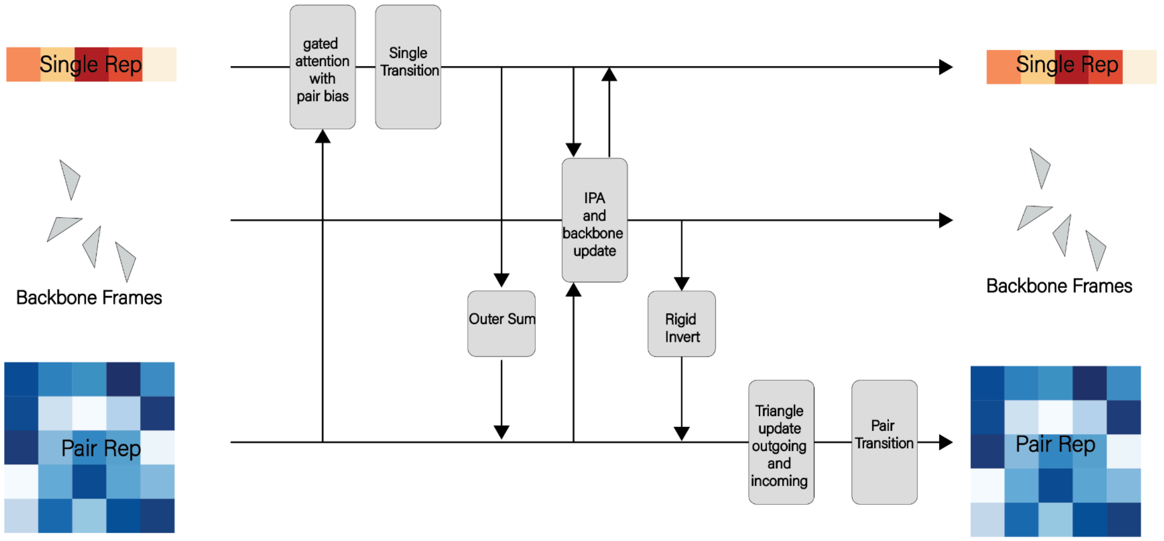

Figure S1. Detailed architecture of TriFlow module showing the information processing and exchange between the three tracks.

bioRxiv preprint doi: https://doi.org/10.64898/2025.11.30.691458; this version posted December 2, 2025. The copyright holder for this preprint (which was not certified by peer review) is the author/funder, who has granted bioRxiv a license to display the preprint in perpetuity. It is made available under aCC-BY 4.0 International license.

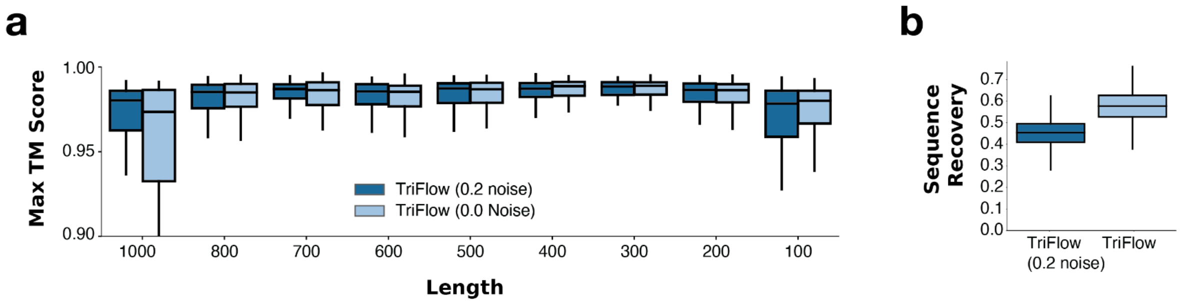

Figure S2. Performance comparison between using noise and not using noise in backbones during sequence generation by TriFlow. **a)** Refoldability of designed sequences for Relaxed sequence optimization (RSO) generated backbone structures; **b)** sequence recovery in the ProteinMPNN test set.

bioRxiv preprint doi: https://doi.org/10.64898/2025.11.30.691458; this version posted December 2, 2025. The copyright holder for this preprint (which was not certified by peer review) is the author/funder, who has granted bioRxiv a license to display the preprint in perpetuity. It is made available under aCC-BY 4.0 International license.

TriFlow (0.2 noise)

Range	Threshold	0.1	0.2	0.3	0.4	0.5	0.6	0.7	0.8	0.9	1.0
long	L	0.472	0.471	0.480	0.482	0.495	0.497	0.500	**0.501**	0.499	0.498
long	L/2	0.647	0.645	0.652	0.666	0.680	0.690	0.704	0.711	0.726	**0.736**
long	L/5	0.717	0.728	0.741	0.746	0.755	0.767	0.786	0.797	0.824	**0.828**
medium	L	0.192	0.194	0.192	0.190	0.193	0.190	0.194	0.196	0.195	**0.196**
medium	L/2	0.336	0.343	0.336	0.337	0.338	0.339	0.341	**0.345**	0.339	0.341
medium	L/5	0.621	0.633	0.636	0.655	0.655	0.657	0.661	0.654	0.662	**0.668**
short	L	0.158	**0.159**	0.157	0.156	0.158	0.156	0.155	0.157	0.156	0.158
short	L/2	0.267	0.268	0.266	0.268	**0.270**	0.264	0.267	0.264	0.263	0.269
short	L/5	0.503	0.529	0.523	0.543	0.543	0.547	**0.557**	0.543	0.554	0.552
Diversity		1.181	1.247	1.344	1.467	1.604	1.758	1.911	2.065	2.213	2.358

TriFlow (0.0 noise)

Range	Threshold	0.1	0.2	0.3	0.4	0.5	0.6	0.7	0.8	0.9	1.0
long	L	0.141	0.158	0.204	0.235	0.269	0.282	0.293	0.303	0.308	0.312
long	L/2	0.221	0.247	0.328	0.387	0.445	0.467	0.485	0.504	0.515	0.530
long	L/5	0.341	0.376	0.527	0.589	0.648	0.669	0.686	0.705	0.715	0.729
medium	L	0.076	0.072	0.090	0.098	0.107	0.110	0.117	0.119	0.124	0.130
medium	L/2	0.120	0.120	0.153	0.165	0.187	0.185	0.199	0.205	0.210	0.219
medium	L/5	0.215	0.237	0.298	0.341	0.386	0.391	0.413	0.411	0.424	0.447
short	L	0.078	0.07[illegible]	0.084	0.091	0.096	0.102	0.106	0.109	0.113	0.114
short	L/2	0.102	0.102	0.118	0.140	0.148	0.164	0.170	0.172	0.181	0.178
short	L/5	0.178	0.192	0.235	0.266	0.308	0.326	0.324	0.345	0.362	0.374
Diversity		0.604	0.653	0.756	0.877	1.009	1.145	1.283	1.418	1.551	1.681

MPNN

Range	Threshold	0.1	0.2	0.3	0.4	0.5	0.6	0.7	0.8	0.9	1.0
long	L	0.268	0.319	0.355	0.382	0.403	0.423	0.441	0.447	0.450	0.445
long	L/2	0.418	0.502	0.556	0.592	0.643	0.660	0.682	0.698	0.699	0.697
long	L/5	0.616	0.692	0.716	0.744	0.769	0.774	0.784	0.802	0.813	0.815
medium	L	0.109	0.122	0.139	0.142	0.149	0.155	0.161	0.174	0.177	0.175
medium	L/2	0.183	0.208	0.234	0.247	0.255	0.268	0.277	0.294	0.303	0.302
medium	L/5	0.352	0.415	0.455	0.493	0.522	0.539	0.560	0.591	0.591	0.574
short	L	0.088	0.100	0.107	0.114	0.124	0.131	0.141	0.144	0.146	0.147
short	L/2	0.127	0.155	0.176	0.186	0.199	0.219	0.229	0.237	0.245	0.241
short	L/5	0.233	0.276	0.347	0.365	0.393	0.435	0.451	0.470	0.476	0.480
Diversity		0.731	0.868	1.051	1.261	1.492	1.735	1.992	2.255	2.515	2.763

ESM-IF

Range	Threshold	0.1	0.2	0.3	0.4	0.5	0.6	0.7	0.8	0.9	1.0
long	L	0.048	0.060	0.088	0.126	0.157	0.174	0.181	0.187	0.195	0.199
long	L/2	0.068	0.090	0.141	0.214	0.270	0.300	0.311	0.318	0.329	0.332
long	L/5	0.111	0.151	0.257	0.389	0.491	0.556	0.574	0.596	0.619	0.624
medium	L	0.054	0.056	0.060	0.075	0.083	0.091	0.090	0.098	0.100	0.102
medium	L/2	0.055	0.065	0.083	0.116	0.135	0.148	0.149	0.166	0.162	0.165
medium	L/5	0.074	0.107	0.152	0.216	0.261	0.293	0.300	0.329	0.322	0.334
short	L	0.066	0.065	0.067	0.071	0.080	0.083	0.085	0.093	0.094	0.098
short	L/2	0.061	0.060	0.076	0.098	0.112	0.119	0.130	0.137	0.144	0.150
short	L/5	0.071	0.075	0.120	0.175	0.211	0.243	0.255	0.272	0.272	0.295
Diversity		0.296	0.473	0.647	0.821	0.994	1.162	1.323	1.477	1.622	1.757

Figure S3. Benchmarking contact prediction across sequence design models. Contact prediction accuracy was evaluated for TriFlow (with and without backbone noise), ProteinMPNN, and ESM-IF. Precision was computed for the top L, L/2, and L/5 predicted contacts across short [6-12), medium-[12-24), and long-range [>24) sequence separations, as well as across model temperature parameters ranging from 0.1 to 1. Diversity is calculated using Shannon's entropy on the generated profiles.

bioRxiv preprint doi: https://doi.org/10.64898/2025.11.30.691458; this version posted December 2, 2025. The copyright holder for this preprint (which was not certified by peer review) is the author/funder, who has granted bioRxiv a license to display the preprint in perpetuity. It is made available under aCC-BY 4.0 International license.

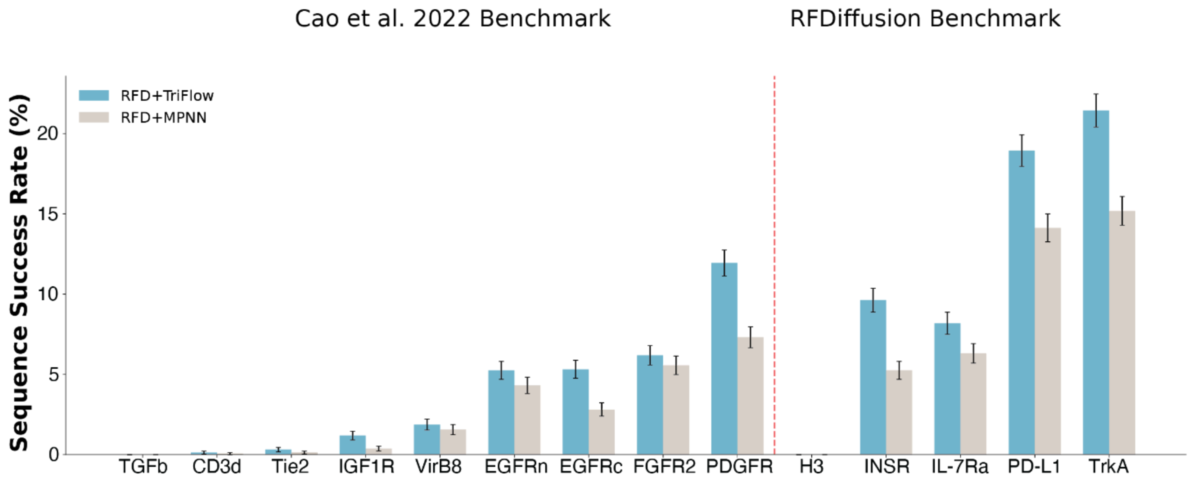

Figure S4. Sequence success rate across target structures. Sequence success rate was defined as the proportion of sequences that passed AlphaFold3 evaluation out of the total 1,600 sequences generated per target. The comparison shows the success rates of sequences generated by TriFlow and ProteinMPNN on backbones produced by RFdiffusion.

bioRxiv preprint doi: https://doi.org/10.64898/2025.11.30.691458; this version posted December 2, 2025. The copyright holder for this preprint (which was not certified by peer review) is the author/funder, who has granted bioRxiv a license to display the preprint in perpetuity. It is made available under aCC-BY 4.0 International license.

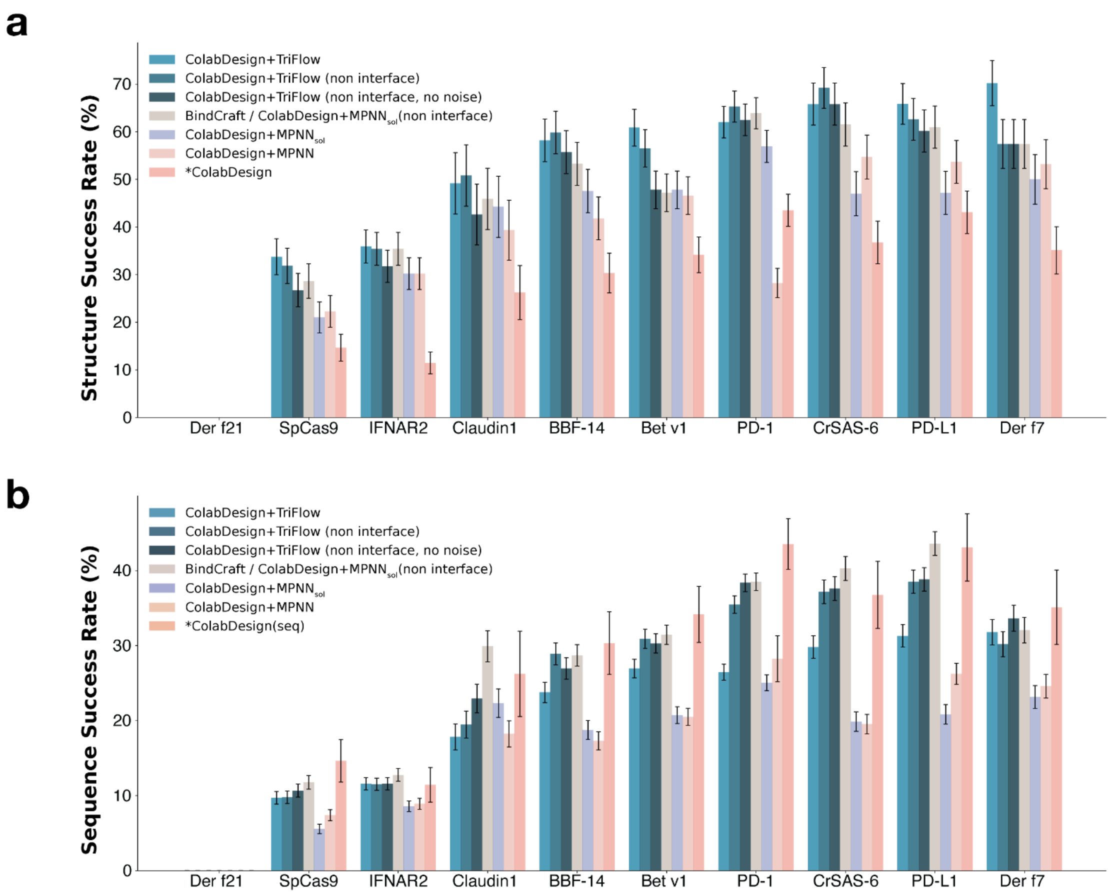

Figure S5. Structure and Sequence success rates for ColabDesign binders with sequence design model ablations. a) Structure success rate comparison across different sequence design strategies. For TriFlow, we evaluated three modes: full binder redesign, redesign restricted to non-interface residues, and sequence design without added structural noise. For ProteinMPNN, analogous ablations were performed, including the BindCraft pipeline (SolubleMPNN redesigning only non-interface residues), full binder redesign with SolubleMPNN, and full redesign with ProteinMPNN. The ColabDesign sequence represents the baseline sequence obtained directly from backbone generation without any redesign. b) Sequence success rates under the same ablation settings for TriFlow and ProteinMPNN.

bioRxiv preprint doi: https://doi.org/10.64898/2025.11.30.691458; this version posted December 2, 2025. The copyright holder for this preprint (which was not certified by peer review) is the author/funder, who has granted bioRxiv a license to display the preprint in perpetuity. It is made available under aCC-BY 4.0 International license.

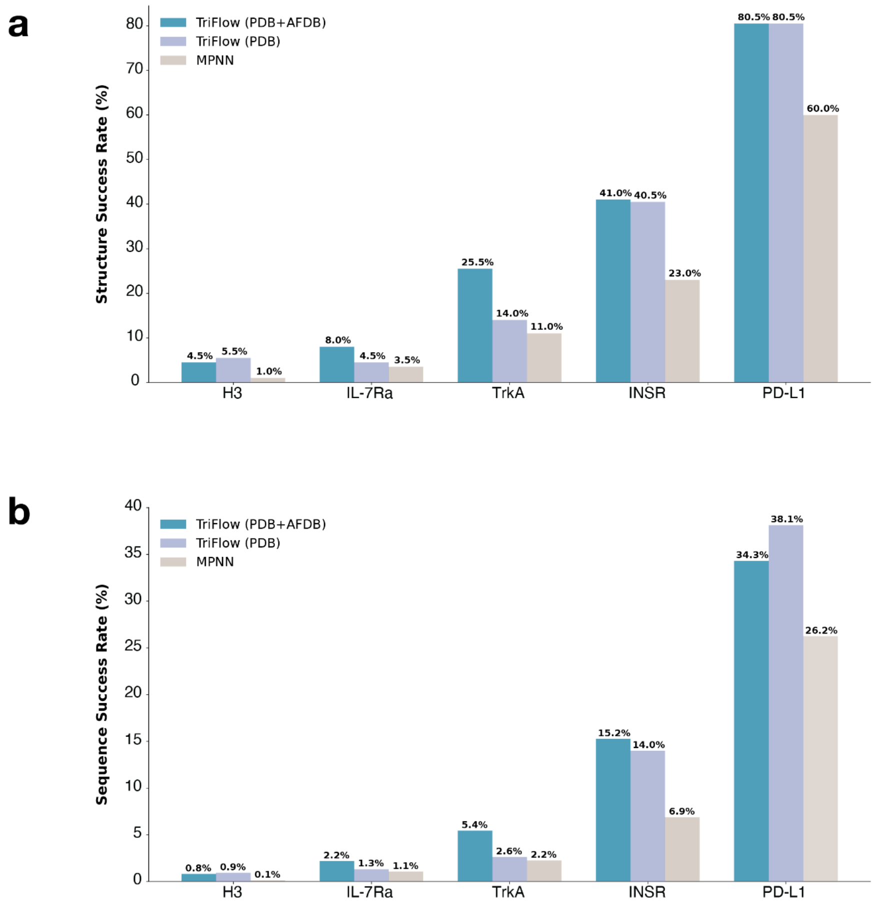

Figure S6. Structure and sequence success rate comparison across training datasets and models. Comparison of structure and sequence success rates for TriFlow trained on the PDB dataset, TriFlow trained on AFDB, and ProteinMPNN, evaluated on RFdiffusion-generated binder backbones. Structure predictions were performed using AlphaFold2 in single-sequence mode with an initial target template provided.

bioRxiv preprint doi: https://doi.org/10.64898/2025.11.30.691458; this version posted December 2, 2025. The copyright holder for this preprint (which was not certified by peer review) is the author/funder, who has granted bioRxiv a license to display the preprint in perpetuity. It is made available under aCC-BY 4.0 International license.

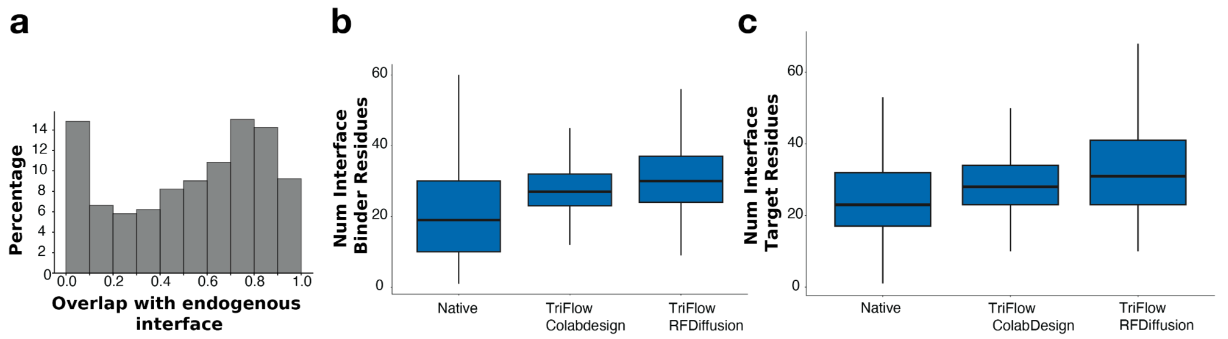

Figure S7. Interface composition and overlap between designed and native binders. a) Distribution of interface overlap between designed binders and their corresponding endogenous (native) interfaces, expressed as the fraction of shared interface residues for ColabDesign binders. b) Comparison of the number of interface residues on the binder side across native complexes, TriFlow+ColabDesign binders, and TriFlow+RFdiffusion binders. c) Comparison of the number of interface residues on the target side across the same sets of structures. Together, these analyses assess how closely designed interfaces recapitulate native interface geometry and residue composition.

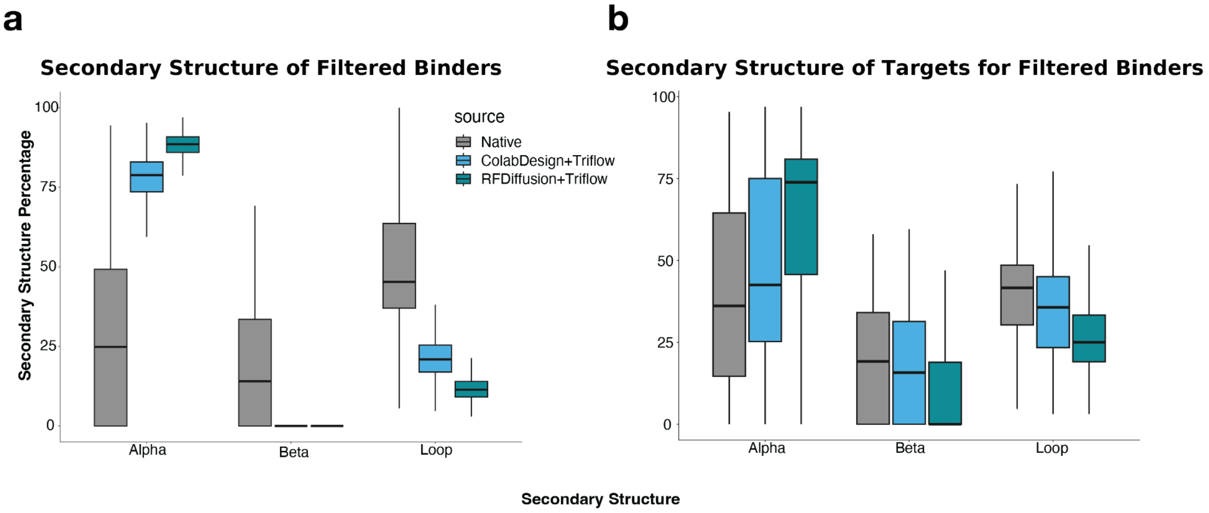

Figure S8. Secondary structure composition of filtered binders and their targets. a) Comparison of secondary structure content among filtered binders generated by TriFlow with ColabDesign and RFdiffusion backbones, relative to native binders. The analysis shows the proportion of alpha-helical, beta-strand, and loop regions within each binder. b) Secondary structure composition of the corresponding target proteins for the same set of filtered binders. This comparison highlights how designed binders differ from native counterparts in their secondary structure distribution while maintaining compatibility with the target interface.

bioRxiv preprint doi: https://doi.org/10.64898/2025.11.30.691458; this version posted December 2, 2025. The copyright holder for this preprint (which was not certified by peer review) is the author/funder, who has granted bioRxiv a license to display the preprint in perpetuity. It is made available under aCC-BY 4.0 International license.

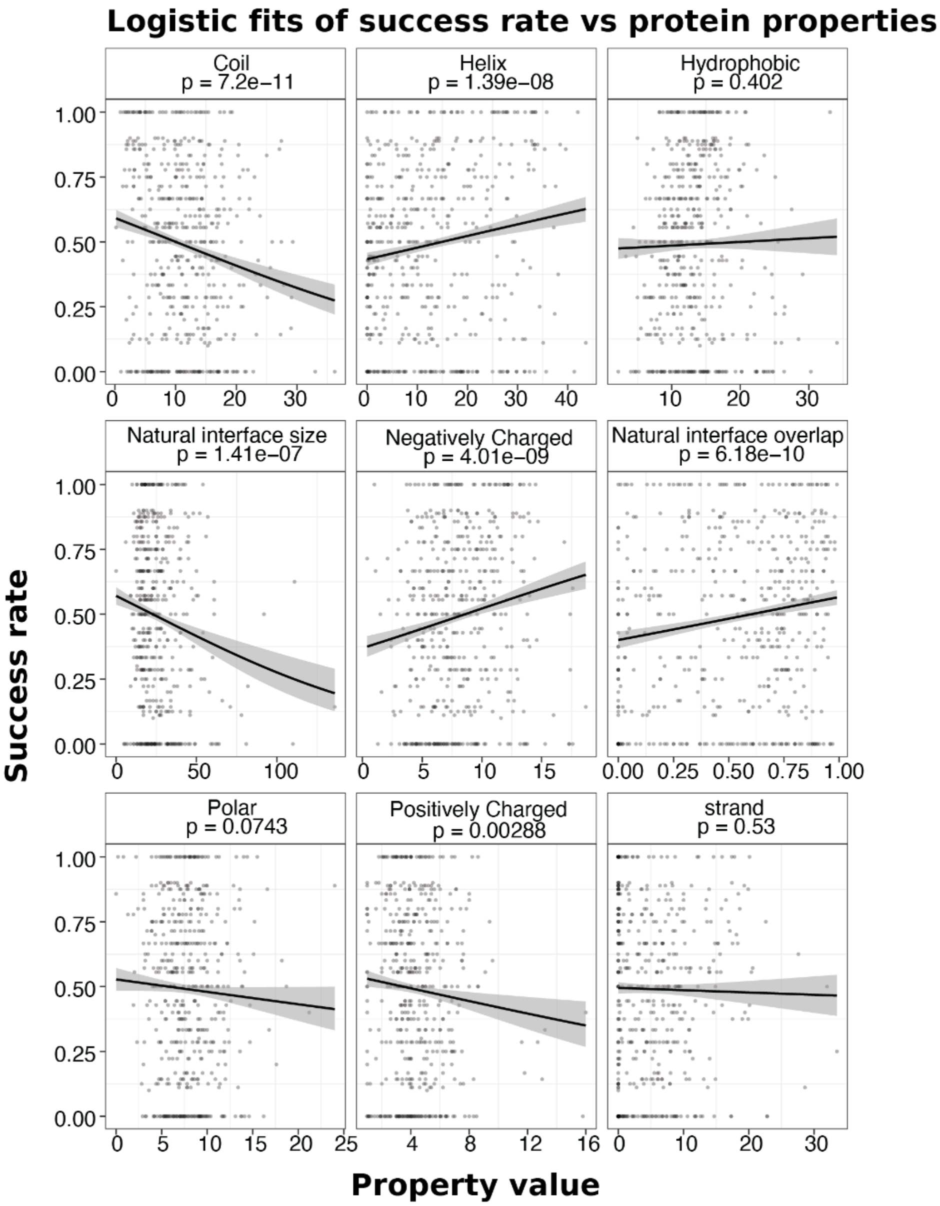

Figure S9. Logistic regression analysis of structural and physical properties influencing design success. Logistic regression models show how both structural and physical features of the target structure correlate with structure success rate. Structural features include secondary structure composition, interface size, and interface overlap between natural and designed binders. Physical properties influenced by the sequence, such as charge distribution and the balance of hydrophobic and polar groups.

bioRxiv preprint doi: https://doi.org/10.64898/2025.11.30.691458; this version posted December 2, 2025. The copyright holder for this preprint (which was not certified by peer review) is the author/funder, who has granted bioRxiv a license to display the preprint in perpetuity. It is made available under aCC-BY 4.0 International license.

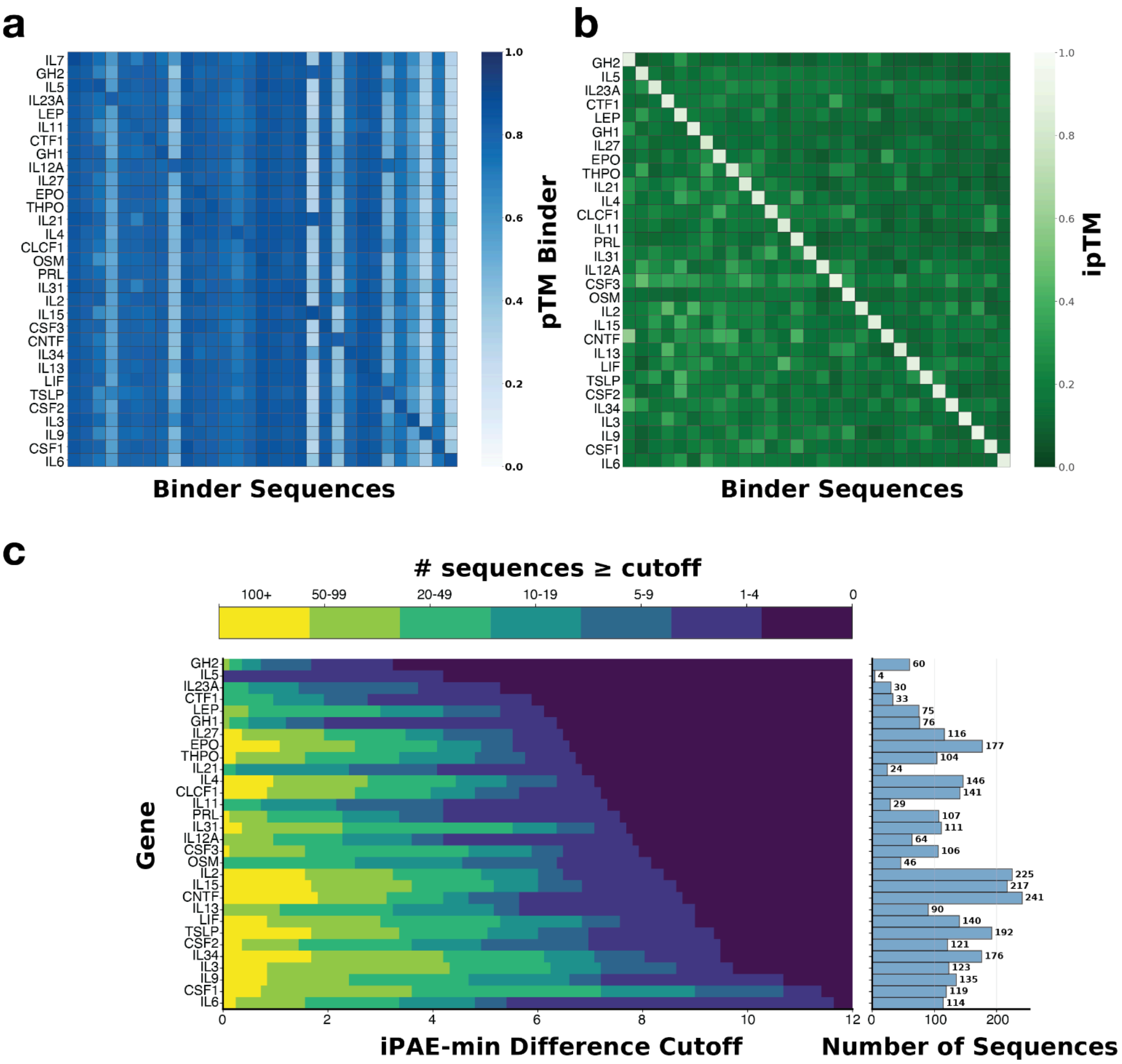

Figure S10. Confidence metrics for generating specific cytokine binders. a) Heatmap of predicted TM-scores showing the structural confidence of the best binder for each cytokine target (on-target) and its corresponding off-target predictions against other cytokines. b) Comparison of interface predicted TM-scores for on-target versus off-target predictions, demonstrating clear separation between specific and non-specific interactions. c) Number of designable sequences available across varying iPAE-min difference cutoffs, where the iPAE-min difference quantifies the gap between the best on-target iPAE-min score and the next-best (lowest) off-target score. Although the metric tracks the best binder per target, multiple sequences meet confidence thresholds at different cutoffs. The histogram (right) summarizes the total number of designable sequences based on AlphaFold3 confidence metrics.

bioRxiv preprint doi: https://doi.org/10.64898/2025.11.30.691458; this version posted December 2, 2025. The copyright holder for this preprint (which was not certified by peer review) is the author/funder, who has granted bioRxiv a license to display the preprint in perpetuity. It is made available under aCC-BY 4.0 International license.

Algorithm 1 Masking Training Loop

```
Set S = 21                                        ▷ number of states: 20 amino acids + mask
Set mask_index = S − 1
Let D denote the sequence length.
for each sequence x_1 and backbone bb_1 in dataset do
                                                  ▷ x_1 has shape (D)
    optimizer.zero_grad()
    u ∼ Uniform(0, 1)
    if u < 0.5 then
        bb ← bb_1 + N(0, 0.2^2)
    else
        bb ← bb_1
    end if
    t ∼ Uniform(0, 1)
    x_t ← x_1
    x_t[rand(D) < (1 − t)] ← mask_index
    logits ← model(x_t, bb, t)                    ▷ (D, 20)
    residue_mask ← 1_D
    residue_mask[x_t ≠ mask_index] ← 0
    per_token_loss ← CrossEntropy(logits, x_1)
    masked_loss ← per_token_loss ⊙ residue_mask
    loss ← Σ masked_loss / (Σ residue_mask + ϵ)
    loss.backward()
    optimizer.step()
end for
```

Figure S11. Pseudocode for training loop.

Algorithm 2 Masking Sampling Loop

```
Initialize t = 0.0, Δt = 0.001
Set number of states S = 21
Set sequence length D
Set mask_index = 20
x_t ← 20 · 1_D
while t < 1.0 do
    logits ← model(x_t, t)                        ▷ (D, 20)
    x_1^probs ← softmax(logits)                   ▷ (D, 20)
    x_1 ∼ Categorical(x_1^probs)                  ▷ sample (D)
    will_unmask ← rand(D) < Δt/(1−t)
    will_unmask ← will_unmask ⊙ (x_t = 20)        ▷ only unmask masked
positions
    x_t[will_unmask] ← x_1[will_unmask]
    t ← t + Δt
end while
return x_t
```

Figure S12. Pseudocode for inference loop.

bioRxiv preprint doi: https://doi.org/10.64898/2025.11.30.691458; this version posted December 2, 2025. The copyright holder for this preprint (which was not certified by peer review) is the author/funder, who has granted bioRxiv a license to display the preprint in perpetuity. It is made available under aCC-BY 4.0 International license.

Table S1. Configuration for running the binder design benchmarks for RFdiffusion and BindCraft

Target	Tool	Input PDB	Hotspot	Binder Size
PD-1	BindCraft	AF2 prediction, trimmed to 32-146	64, 126, 129, 133	80-150
PD-L1	BindCraft	AF2 prediction, trimmed to 18-132	54, 56, 66, 115	65-155
IFNAR2	BindCraft	2LAG, trimmed to 8-110	52, 80, 82, 84, 96, 98	60-175
Claudin1	BindCraft	AF2 prediction of CLN1-14	31, 46, 55, 152	80-175
BBF-14	BindCraft	9HAG	none	70-250
CrSAS-6	BindCraft	AF2 prediction, trimmed to 15-160	none	90-200
Der f7	BindCraft	AF2 prediction with 3UV1 template, trimmed to 18-213	132	70-185
Der f21	BindCraft	AF2 prediction with 5YNY template, trimmed to 25-136	34	70-185
Bet v1	BindCraft	AF2 prediction	24	70-185
SpCas9	BindCraft	AF2 prediction with 4ZT0 template, trimmed to 96-174 + 306-446	360	70-150
TGFb	RFdiffusion	AF2 prediction	B14,B63,B66,B67	40-100
CD3d	RFdiffusion	AF2 prediction	A6,A11,A31,A32	40-100
Tie2	RFdiffusion	AF2 prediction	A127,A137,A138,A171	40-100
IGF1R	RFdiffusion	AF2 prediction	A52,A78,A87,A108	40-100
Virb8	RFdiffusion	AF2 prediction	A43,A47,A58,A59	40-100
EGFRn	RFdiffusion	AF2 prediction	A8,A9,A95,A99	40-100
EGFRc	RFdiffusion	AF2 prediction	A72,A102,A108,A128	40-100
FGFR2	RFdiffusion	AF2 prediction	A4,A48,A87,A89	40-100
PDGRF	RFdiffusion	AF2 prediction	A116,A120,A138,A141	40-100
H3	RFdiffusion	5VLI	B521, B545, B552	40-120
INSR	RFdiffusion	4ZXB	E64, E88, E96	40-120
IL-7Ra	RFdiffusion	3DI3	B58, B80, B139	50-120
PD-L1	RFdiffusion	5O45	A56, A115, A123	50-120
TrkA	RFdiffusion	1WWW	X294, X296, X333	50-120
